# Applications of natural language processing and large language models in sports injury assessment and rehabilitation decision-making: a scoping review

**DOI:** 10.3389/fmed.2026.1866874

**Published:** 2026-07-06

**Authors:** Hao Wang, Youxian Liu, Lirong Hu, Xiangjin Wang

**Affiliations:** 1 First Clinical Medical College, Fujian University of Traditional Chinese Medicine, Fuzhou, China; 2 School of Rehabilitation Medicine, Affiliated Rehabilitation Hospital of Fujian University of Traditional Chinese Medicine, Fujian University of Traditional Chinese Medicine, Fuzhou, China; 3 Hospital of Chengdu University of Traditional Chinese Medicine, Chengdu, China

**Keywords:** benchmarking, clinical decision support, generative artificial intelligence, large language models, natural language processing, patient education, sports injuries, sports rehabilitation

## Abstract

**Objective:**

This study aims to provide a scoping review of the current applications of natural language processing (NLP) and large language models (LLMs) in the assessment of sports injuries and rehabilitation decision-making, with the goal of identifying the technical methods, data sources, target populations, key findings, and knowledge gaps in existing research, and to provide evidence-based guidance for clinical practice.

**Methods:**

We followed the Joanna Briggs Institute (JBI) scoping review framework and the PRISMA-ScR guidelines to search the PubMed, Scopus, and Web of Science Core Collection databases (up to March 31, 2026). We included studies on sports injury and rehabilitation that utilized NLP or LLMs to process unstructured text. Two researchers independently screened the studies and extracted data; disagreements were resolved through discussion or third-party arbitration. Study quality was assessed using MINimum Information for Medical AI Reporting (MINIMAR).

**Results:**

A total of 27 studies were included. The majority of the studies originated from the United States (37.0%). The primary research types were algorithm evaluation and benchmarking. Healthcare professionals were the main target population. The text data sources consisted primarily of simulated/synthetic question-answering scenarios. The included studies faced challenges such as readability issues and AI hallucinations. The overall compliance rate with the MINIMAR report quality assessment was 61.70%.

**Conclusion:**

LLMs have shown great potential as tools for sports injury assessment and rehabilitation decision-making. However, they still have significant shortcomings in terms of readability, hallucination control, patient perspective, methodological reporting, and geographic coverage. Future efforts should be grounded in the sports field, with a focus on athletes and team physicians, to improve model reliability, bridge geographic gaps, elevate research standards, and facilitate the transition from baseline assessment to clinical application.

## 1 Introduction

In the sports injury assessment and rehabilitation decision-making, traditional clinical pathways primarily rely on standard quantitative data, such as biomechanical sensor data, medical imaging, and physiological and biochemical indicators. However, these parameters often fail to fully capture the complex characteristics of sports injuries and the rehabilitation process. A wealth of critical information regarding injury mechanisms, disease progression, and patients' psychological readiness for return to play is recorded in the form of unstructured free-text in electronic health records, clinical notes by physicians or therapists, and patient self-reports during consultations (1–3). Effectively extracting and quantifying these deep semantic features has become key to overcoming current bottlenecks in sports medicine (4).

With the advancement of artificial intelligence (AI), natural language processing (NLP) technologies—particularly large language models (LLMs) based on the Transformer architecture, such as ChatGPT and Gemini—have evolved from basic text classification to the analysis of complex medical descriptions. Currently, LLMs are being widely applied to extract structured injury features, construct knowledge graphs, and even generate personalized clinical recommendations and exercise prescriptions (5, 6). However, despite an explosive growth in related research, significant knowledge gaps remain in this interdisciplinary field: existing literature lacks systematic mapping across different text sources; simultaneously, the adherence of clinical reports on these AI algorithms to established standards [such as the Medical AI Reporting (MINIMAR) criteria] has not been rigorously examined (7).

In light of this, this scoping review aims to comprehensively map the application landscape of NLP and LLMs in sports injury assessment and rehabilitation decision-making, evaluate their methodological characteristics and clinical efficacy, and provide evidence-based guidance for future real-world clinical practice.

## 2 Materials and methods

This study conducted a scoping review based on the methodology of the Joanna Briggs Institute (JBI) and the PRISMA-ScR guidelines (8). The study protocol was registered on the Open Science Framework (OSF) at http://osf.io, at the following link: https://osf.io/jbd8v?view_only=c9efc9eee79e4d1992bb9dc65e60aa76.

### 2.1 Identifying the research questions

Based on a comprehensive literature review and group discussions, the main research questions of this study are defined as follows: (1) How can NLP technologies, such as LLMs, be effectively utilized to analyze “unstructured text data” in sports injury assessment and rehabilitation decision-making? (2) What challenges does this technology currently face in its transition to real-world clinical practice?

### 2.2 Search strategy

The search strategy combined Medical Subject Headings (MeSH), free-text terms, and Boolean operators. No language restrictions were applied during the search. The search query primarily consisted of the intersection (AND) of three logical modules: (1) artificial intelligence and computational linguistics; (2) exercise and sports contexts; and (3) sports injuries and rehabilitation. Synonyms within each module were combined using OR, while the three modules were combined using AND. To ensure the comprehensiveness of the literature, no strict lower limit was set for the publication year; the search was conducted up to March 31, 2026. In addition, forward citation searching was conducted to identify relevant studies that were not found through database searches alone (9). For details, see Table 1. The initial search strategy included the term “Tuina” as part of the rehabilitation module to capture studies involving traditional manual therapy approaches that might intersect with NLP/LLM applications. However, this term yielded no relevant records in PubMed, Scopus, or WoScc. In the interest of parsimony and to improve the specificity of the search strategy, “Tuina” was removed from the final search string presented in Table 1. Its removal did not affect the search yield, as the term generated zero eligible records.

**Table 1:** Search strategy.

Eligibility criteria	
Inclusion criteria	Exclusion criteria
•Population: athletes or sportspeople; healthcare professionals involved in the assessment and triage of sports injuries and rehabilitation •Concept: it employs algorithms such as natural language processing, computational linguistics, text mining, and large language models to process unstructured text data. •Context: sports science, sports medicine, sports injury monitoring, conservative treatment, and physical rehabilitation settings.	•Studies whose objective does not correspond to the research question. •Literature published in languages other than English; •Non-peer-reviewed studies, including conferenceabstracts/proceedings, preprints, letters, editorials, and expert reviews •Studies that use only traditional machine learning for numerical prediction; •Pure animal experimental studies that do not explicitly involve unstructured text mining
Search strategies Databases	
PubMed	**Health Science Descriptors (DeCS) and Medical Subject Headings (MeSH) terms used** (“Artificial Intelligence”[Mesh] OR “Natural Language Processing”[Mesh] OR “Machine Learning”[Mesh] OR “Artificial Intelligence”[Title/Abstract] OR “Natural Language Processing”[Title/Abstract] OR “NLP”[Title/Abstract] OR “Text Mining”[Title/Abstract] OR “Computational Linguistics”[Title/Abstract] OR “Semantic Network^*^”[Title/Abstract] OR “Discourse Analysis”[Title/Abstract] OR “Critical Discourse Analysis”[Title/Abstract] OR “Large Language Model^*^”[Title/Abstract] OR “LLM”[Title/Abstract] OR “LLMs”[Title/Abstract] OR “Generative AI”[Title/Abstract] OR “ChatGPT”[Title/Abstract]) AND ((“Athletic Injuries”[Mesh] OR “Sports Medicine”[Mesh] OR “Return to Sport”[Mesh] OR “Sports Injur^*^”[Title/Abstract] OR “Athletic Injur^*^”[Title/Abstract] OR “Sports Medicine”[Title/Abstract] OR “Return to play”[Title/Abstract] OR “Return to sport^*^”[Title/Abstract]) OR ((“Sports”[Mesh] OR “Athletes”[Mesh] OR “Sport^*^”[Title/Abstract] OR “Athlete^*^”[Title/Abstract] OR “Athletic^*^”[Title/Abstract]) AND (“Musculoskeletal Pain”[Mesh] OR “Neuralgia”[Mesh] OR “Chronic Pain”[Mesh] OR “Rehabilitation”[Mesh] OR “Physical Therapy Modalities”[Mesh] OR “Musculoskeletal Manipulations”[Mesh] OR “Massage”[Mesh] OR “Musculoskeletal Pain”[Title/Abstract] OR “Neuropathic Pain”[Title/Abstract] OR “Chronic Pain”[Title/Abstract] OR “Rehabilitation”[Title/Abstract] OR “Physical Therapy”[Title/Abstract] OR “Manual Therapy”[Title/Abstract] OR “Massage”[Title/Abstract])))
Scopus	TITLE-ABS-KEY (“Artificial Intelligence” OR “Natural Language Processing” OR “Machine Learning” OR “NLP” OR “Text Mining” OR “Computational Linguistics” OR “Semantic Network^*^” OR “Discourse Analysis” OR “Critical Discourse Analysis” OR “Large Language Model^*^” OR “LLM” OR “LLMs” OR “Generative AI” OR “ChatGPT”) AND (TITLE-ABS-KEY (“Sports Injur^*^” OR “Athletic Injur^*^” OR “Sports Medicine” OR “Return to play” OR “Return to sport^*^”) OR (TITLE-ABS-KEY (“Sport^*^” OR “Athlete^*^” OR “Athletic^*^”) AND TITLE-ABS-KEY (“Musculoskeletal Pain” OR “Neuropathic Pain” OR “Chronic Pain” OR “Rehabilitation” OR “Physical Therapy” OR “Manual Therapy” OR “Massage”)))
Web of science core collection	TS=(“Natural Language Processing” OR “NLP” OR “Text Mining” OR “Computational Linguistics” OR “Semantic Network^*^” OR “Discourse Analysis” OR “Critical Discourse Analysis” OR “Large Language Model^*^” OR “LLM^*^” OR “Artificial Intelligence” OR “Machine Learning” OR “Generative AI” OR “ChatGPT”) AND (TS=(“Sports Injur^*^” OR “Athletic Injur^*^” OR “Sports Medicine” OR “Return to play” OR “Return to sport^*^”) OR (TS=(“Sport^*^” OR “Athlete^*^” OR “Athletic^*^”) AND TS=(“Musculoskeletal Pain” OR “Neuropathic Pain” OR “Chronic Pain” OR “Rehabilitation” OR “Physical Therapy” OR “Manual Therapy” OR “Massage”)))

### 2.3 Inclusion and exclusion criteria

To be included in this review, all evidence must meet the Population, Concept, and Context (PCC) inclusion criteria (8):

Population: professional athletes or recreational exercisers who have sustained sports-related injuries, or healthcare professionals involved in the assessment and triage of sports injuries and rehabilitation.

Concept: explicit application of algorithms such as NLP, computational linguistics, text mining, or LLMs to process unstructured text data.

Context: limited to the fields of sports science, sports medicine, sports injury monitoring, conservative treatment, and physical rehabilitation.

Exclusion Criteria: (1) Literature published in languages other than English; (2) Non-peer-reviewed studies, including conference abstracts/proceedings, preprints, letters, editorials, and expert reviews; (3) Studies that use only traditional machine learning for numerical prediction; and (4) Pure animal experimental studies that do not explicitly involve unstructured text mining.

### 2.4 Literature screening and selection process

The literature screening process consisted of three stages. First, all search results were imported into Zotero, and duplicates were automatically removed. Second, during the initial screening of titles and abstracts, all studies were screened based on predefined keyword expressions to exclude those not involving text mining, those unrelated to sports medicine, or those that were clearly irrelevant. Finally, a rigorous full-text and in-depth abstract evaluation was conducted. Two researchers (WH and LYX) independently reviewed studies based on titles, abstracts, and full texts to determine their relevance; any discrepancies were resolved through discussion or consultation with a third reviewer (WXJ). From the initial 3,063 identified studies, 27 core studies were selected after filtering for topic relevance for data extraction and synthesis.

### 2.5 Data extraction and charting

Two researchers (WH and LYX) independently extracted the data, cross-checked the results, and conducted a comprehensive analysis. Any discrepancies were resolved through consultation with a third researcher (HLR). The extracted data included country, year of publication, first author, study design, target population, sport, injury type, NLP/LLM method, text data source, specific NLP task, key findings, and limitations; see Supplementary Appendix 1 for details. Although 27 studies were included in the scoping review, only 23 empirical studies were eligible for reporting quality assessment using the MINIMAR framework (10). Four studies were excluded from this assessment because they were review or opinion studies for which MINIMAR criteria are not readily applicable.

## 3 Result

**Figure 1:**
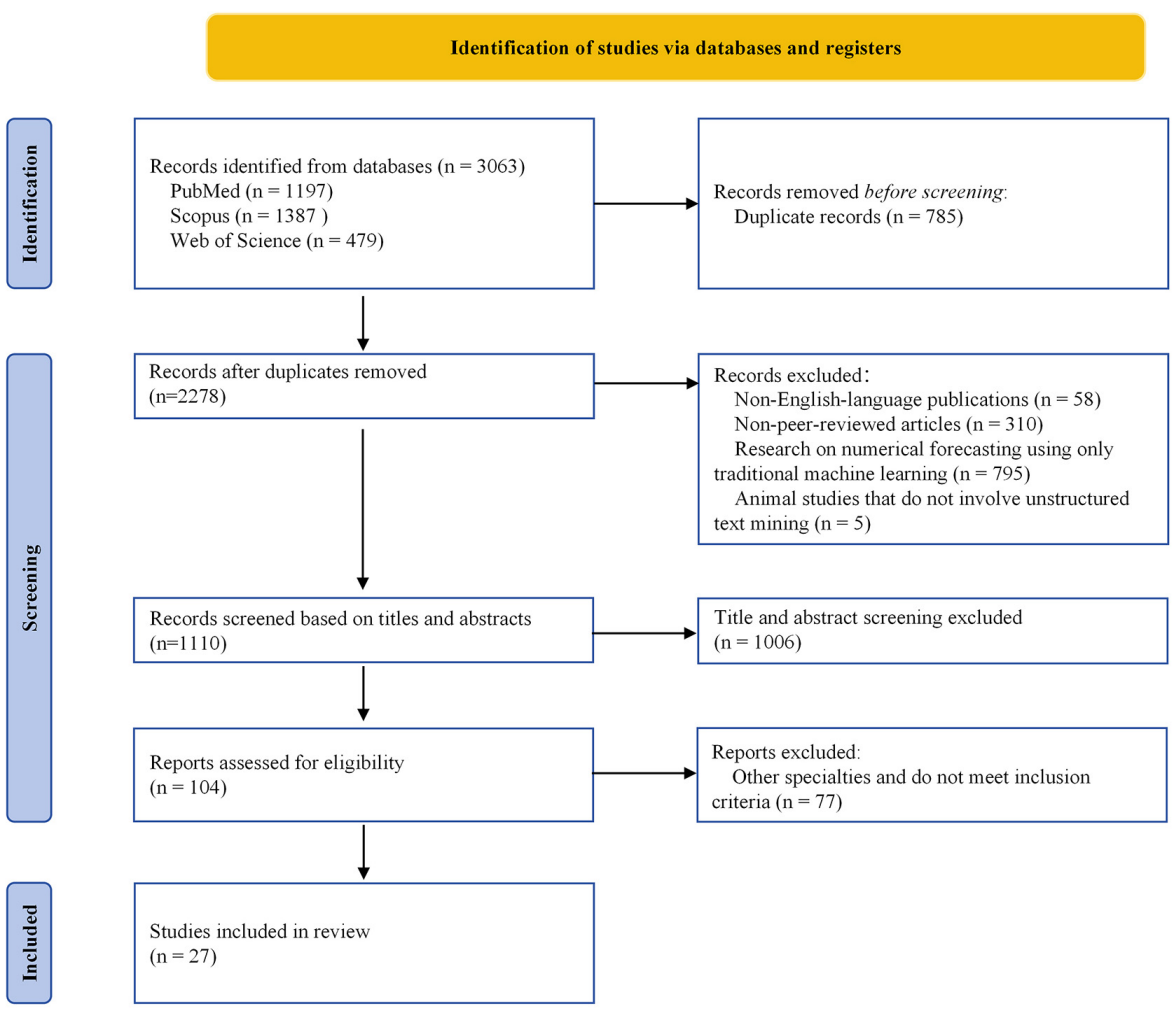
PRISMA-ScR flow diagram of study selection and inclusion process.

The initial search yielded a total of 3,063 studies from PubMed, Scopus, and WoScc (PubMed: 1,197; Scopus: 1,387; WoScc: 479). After deduplication (removing 785 studies), 2,278 studies remained. After screening based on exclusion criteria, 1,110 studies remained. Following a PCC assessment of titles and abstracts combined with exclusion criteria, 1,006 studies were excluded, leaving 104. After manual review, 27 studies were ultimately included. The literature screening process is illustrated in Figure 1.

### 3.1 Overall trends in publishing

#### 3.1.1 Year of publication, country, and type of research

The 27 included studies were published between 2023 and 2026. Specifically, two were published in 2023 (7.4%), six in 2024 (22.2%), 11 in 2025 (40.8%), and eight in 2026 (29.6%). Since 2023, the number of publications in this field has shown a rapid growth trend, peaking in 2025. This aligned with the development trends of LLMs (such as ChatGPT) and indicated that applied research on LLMs in the field of sports injuries and rehabilitation was in a period of rapid development.

**Figure 2:**
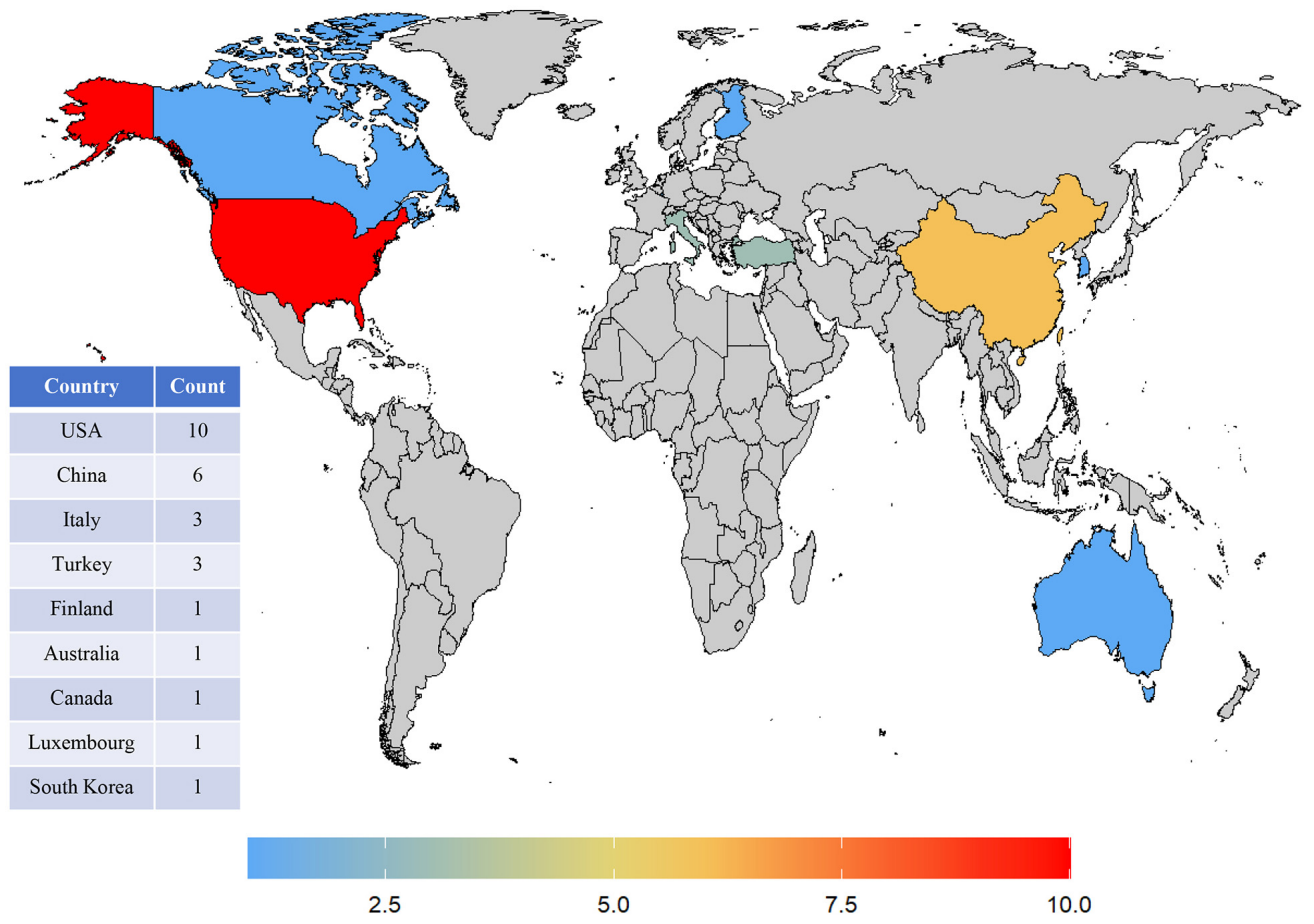
Global distribution of publications.

In terms of the first authors' countries/regions of affiliation (Figure 2), the 27 studies are distributed across eight countries and regions. The United States contributed the most, with a total of 10 studies (37.0%) (11–20); China (including mainland China and Taiwan) followed with six studies (21–26). Italy and Turkey each contributed three studies (27–32); Canada, Australia, Luxembourg, South Korea, and Finland each contributed one paper (33–37). No relevant studies have yet been published from South America, Africa, the Middle East (excluding Turkey), or Southeast Asia.

The study designs of the included literature primarily focused on algorithm evaluation and benchmarking, with a total of 10 studies. In such studies, sports medicine experts typically scored the responses generated by LLMs in a blinded or unblinded manner to assess accuracy, completeness, and readability (11, 14, 16, 17, 21–23, 32, 36, 37). There were five cross-sectional studies, primarily used to compare the performance of different LLMs vs. traditional search engines (18–20, 25, 31). Eight studies fell into both the algorithm evaluation and benchmarking category and the cross-sectional study category (12, 13, 24, 26, 28, 30, 33, 35); there were two opinion pieces (27, 34) and two reviews (15, 29).

#### 3.1.2 Target population

Depending on the primary users, the target populations in the included studies can be divided into two main categories: patients (including athletes and the general population engaged in physical activity) and healthcare professionals. A total of six studies focused on patients as the primary target population (12, 19, 20, 25, 28, 32). These studies focused on the “readability,” “empathy,” and “patient education” value of the generated content, aiming to help patients alleviate preoperative anxiety or improve their understanding of treatment plans.

There were 17 studies in which healthcare professionals served as evaluators or the primary target users (11, 13–18, 21, 24, 26, 27, 30, 31, 34–37). Healthcare professionals (including orthopedic surgeons, sports medicine physicians, physical therapists, residents, and researchers) play a dual role: they are both the target users of the advanced professional functions of LLMs (such as diagnostic assistance, triage, and literature extraction) and the evaluators of the accuracy and clinical safety of model outputs.

Four studies included both patients and healthcare professionals (22, 23, 29, 33). These studies explored how LLMs can serve as a bridge to meet the needs of both groups. The models can automatically adjust the level of professional detail in their responses based on the user's role or convert subjective patient experiences into quantifiable clinical intervention metrics that are actionable by healthcare providers.

#### 3.1.3 Distribution of sports and injury types

Distribution of sports: among the included studies, those that did not specify a particular sport were the most numerous, totaling 25 (11–15, 17–35, 37). These studies did not limit their context to specific sports but instead focused on general musculoskeletal injuries or rehabilitation scenarios. Only a very small number of studies focused on the epidemiological characteristics of specific sports or specific movement patterns, evaluating AI models within the unique contexts of those sports, including soccer, squash, badminton, and tennis (16, 36).

Distribution of injuries and clinical conditions: there were 14 comprehensive studies on sports injuries (11, 15, 17, 19–23, 27, 29–31, 34, 37). These studies are not limited to a single anatomical site but test the ability of LLMs to handle a wide range of musculoskeletal issues, multi-joint systems, or comprehensive clinical knowledge. This includes comprehensive evaluations of the Orthopedic Intern and Resident Examination (OITE), the development of systemic exercise prescriptions, basic neuromuscular reflex assessments, the Functional Movement Screen (FMS), and cross-disciplinary questions on biologics and routine orthopedic surgery.

There were 13 studies that addressed specific injuries (12–14, 16, 18, 24–26, 28, 32, 33, 35, 36). These studies focus on highly specific anatomical structures or individual clinical conditions, providing an in-depth evaluation of LLMs' capabilities in diagnosis, triage, and patient education regarding specific sports injuries. Among them, eight studies address knee injuries, three address shoulder and elbow injuries, and one each addresses plantar fasciitis and craniofacial injuries.

### 3.2 Text data source

The sources of text data used in the included studies were divided into two main categories: real-world clinical text and simulated/synthetic Q&A.

Real clinical text refers to free-text data derived directly from clinical practice or clinical settings. A total of six studies fall into this category, including clinical notes from electronic health records, diagnostic/imaging reports, pre-visit free-text questionnaires, surgical records, athlete self-reported feedback, news and social media texts, injury descriptions from the NEISS database, and text metadata accompanying electromyography waveform images (17, 18, 21–23, 36).

Simulated/synthetic Q&A refers to standardized questions manually constructed by researchers based on guidelines, consensus documents, exam question banks, or common online inquiries, comprising a total of 17 studies (11–14, 16, 19, 20, 24–26, 28, 30–33, 35, 37). These include questions adapted from clinical practice guidelines, common search engine queries, Delphi consensus questions, OITE exam questions, simulated clinical case histories, and scenarios.

There are significant differences in research conclusions between the two types of data sources. Studies based on real clinical text focus on predictive tasks, such as recommendations for imaging examinations and surgical procedures, and more closely reflect actual clinical workflows, but are fewer in number. In contrast, simulated Q&A studies are primarily used to evaluate the accuracy of LLM knowledge, consistency with guidelines, and the readability of patient education content; they are easier to standardize and compare but may overestimate LLM performance in real-world scenarios.

### 3.3 NLP and LLMs methodologies

#### 3.3.1 The model used

The models included in the literature were categorized into closed-source and open-source models. Researchers primarily utilized the powerful zero-shot natural language understanding and generation capabilities of closed-source commercial models for benchmarking and question-answering tasks. These models include OpenAI (ChatGPT / GPT-3.5 / GPT-4 / GPT-4o series) (11–14, 16, 18–20, 24–26, 28, 30–33, 35, 36), Google (Gemini / the original Bard series) (11, 13, 19, 20, 25, 26, 28), Anthropic (Claude series) (25, 26), as well as Deepseek R1 and Grok3 (19, 32). Open-source LLMs can be deployed locally, support full-parameter fine-tuning, or possess image interpretation capabilities, including the Qwen and Llama series (17, 23, 37). Some studies developed or utilized underlying architectures highly customized for specific medical tasks (21, 22, 26). Additionally, 13 studies employed a multi-model comparison study design, which not only tested the effectiveness of individual models but also conducted a comparative evaluation of the strengths and weaknesses of different underlying algorithms (such as those from OpenAI, Google, and Anthropic) in specific medical tasks.

#### 3.3.2 Technical methods

The technical methods employed in the included studies were categorized into three levels:

Six studies utilized basic NLP methods, primarily relying on dictionaries and rules. These included the use of Linguistic Inquiry and Word Count for sentiment and psycholinguistic feature analysis, the use of TF-IDF for text vectorization, and rule-based calculation of scoring metrics (19, 20, 23, 25, 32, 38). Examples include algorithmic studies that use BLEU/ROUGE metrics to evaluate the quality of Q&A model outputs, as well as LIWC and TF-IDF bag-of-words extraction techniques summarized in reviews of NLP applications in psychological counseling.

Traditional machine learning methods primarily include logistic regression, random forests, support vector machines, and extreme gradient boosting, which are frequently used for clinical triage prediction, injury risk classification, and binary/multiclass classification tasks related to answer accuracy, with a total of three studies (15, 29, 38). A few studies employed Long Short-Term Memory (LSTM) or Convolutional Neural Networks (CNN) as comparison baselines; however, as these are not central to this review, detailed results can be found in the original publications.

Deep learning methods, centered on Transformer-based LLMs, include the fine-tuning of pre-trained models such as BERT, RoBERTa, and XLNet, as well as the use of vision-language models for image-text multimodal analysis (12–15, 17, 18, 21–23, 26, 36–38). The included studies primarily address pre-trained LLMs, parameter-efficient fine-tuning and underlying architecture optimization, visual-language multimodal fusion, and CNNs.

### 3.4 NLP tasks and primary applications

The NLP tasks performed in the included studies were categorized into five major categories. The five categories described below are organized from core to extended, reflecting their proximity to the central research questions defined by the PCC. Categories one and two (clinical decision support and triage; question-answering and patient education) constitute core applications that directly address tasks related to the assessment or rehabilitation of sports injuries. Categories three through five (text information extraction; medical examination and knowledge assessment; multimodal and sentiment analysis) represent extended applications that evaluate foundational clinical knowledge, demonstrate transferable technical capabilities, or provide essential interdisciplinary context; each category contributes necessary evidence toward a comprehensive understanding of the readiness of NLP/LLM for deployment in sports medicine.

Clinical decision support and triage were addressed in 12 studies (13, 14, 16, 18, 22, 24, 26, 29–31, 34, 35). These included generating differential diagnoses, predicting the need for imaging tests, treatment decisions and surgical predictions, guideline compliance reasoning, discussions on management-level decisions, developing rehabilitation plans, and risk stratification.

Question-answering systems and patient education were covered in nine studies (12, 19, 20, 23, 25, 27, 28, 32, 33). Research in this category typically addresses natural-language-based medical queries and generates lay-friendly medical texts for the general public. Its core objective is to bridge the professional information gap between physicians and patients by providing high-quality preoperative preparation guidance, postoperative rehabilitation advice, and knowledge on sports injury prevention.

Text information extraction comprises two studies (15, 36). These tasks focus on automatically identifying, extracting, and reconstructing specific medical, factual, or epidemiological data from vast amounts of unstructured text (such as online news, sports media reports, public databases, or electronic health records). This significantly reduces the manual workload for researchers in injury and disease monitoring and data organization.

Medical examinations and knowledge assessment comprise two studies (11, 37). These studies used questions from the Orthopedic Internship Exam (OITE) to assess the clinical knowledge of LLMs. By having the LLMs answer multiple-choice questions, the study quantitatively evaluated the extent of their knowledge in orthopedics and sports medicine.

Multimodal analysis and sentiment analysis comprise three studies, representing the latest technological frontiers in the field of medical artificial intelligence (17, 21, 38). On the one hand, multimodal analysis breaks through the limitations of text-only data by deeply integrating computer vision (CV), time-series physiological signals, and NLP; on the other hand, sentiment analysis delves into the psychological state of patients underlying the text.

### 3.5 Key findings and limitations

#### 3.5.1 Key findings

The key findings reported in the included literature can be summarized in the following four areas:

(1) LLMs demonstrate moderate to good accuracy in answering questions about sports injuries. Studies indicate that LLMs possess clinical knowledge equivalent to that of a 2nd-year orthopedic resident (11, 33). Regarding adherence to clinical practice guidelines, mainstream models generally scored four or higher (out of five) on responses to AAOS guidelines (13); in the evaluation of guidelines for acute meniscal injuries, the healthcare-specific model OpenEvidence achieved 100% agreement (26).
(2) LLMs show potential in clinical triage and decision support, but carry a risk of error. ChatGPT-4 achieved a 70% agreement rate in initial orthopedic triage (14). By analyzing pre-visit questionnaires and imaging reports, its accuracy in predicting recommended tests and surgeries was 70 and 81%, respectively (18). Furthermore, risk stratification for racquet sports injuries revealed extremely low quality scores for treatment recommendations (1.4/5), indicating that current models lack the ability to independently provide high-quality clinical advice (16).
(3) Patient education content generated by LLMs exhibits good structural integrity but is generally difficult to read. Its Flesch-Kincaid Grade Level (FKGL) far exceeds the sixth-grade standard recommended by the American Medical Association. Multiple studies indicate that text generated by ChatGPT and Gemini typically falls within the reading difficulty range of high school to college level (16, 20). King et al. further confirmed that the three major mainstream models (ChatGPT, Gemini, and Grok) all exhibit extremely high reading difficulty in clinical question-answering tasks (19).
(4) Open-source models and fine-tuning strategies hold significant potential. Research indicates that the performance of open-source vision-language models (VLMs) improves with an increase in parameter scale (up to 72B), reaching a level comparable to that of a 2nd-year resident (37). Regarding underlying fine-tuning techniques, GaLore's full-parameter fine-tuning outperforms traditional LoRA methods in terms of resource consumption, convergence speed, and text quality (23). Additionally, studies have shown that multi-model federation strategies not only significantly improve the accuracy and consistency of complex physiological signal interpretation but also achieve highly interpretable clinical consensus outputs (17).

#### 3.5.2 Limitations

The included literature reports have the following limitations:

(1) Small sample sizes and limited generalizability. Most studies used a limited number of standardized questions, making it difficult to capture the complexity of real-world sports injury clinical settings (12, 32). Furthermore, due to the small sample sizes, the results are difficult to generalize to other types of sports injuries or populations from different cultural backgrounds (14, 18).
(2) Evaluations rely on experts' subjective judgments and lack a patient perspective. Experts' assessments of “content accuracy” are not equivalent to patients' perceptions of “comprehensibility,” “credibility,” and “emotional resonance.” This discrepancy may lead researchers to overestimate the actual clinical value of LLM outputs.
(3) LLMs carry risks of “hallucinations” and outdated information. Multiple studies have reported instances of AI hallucinations in LLMs, particularly when prompts are insufficient or when dealing with areas where evidence is still inconclusive. Hallucinations can be categorized into three types: factual errors, inconsistencies with guidelines, and fabricated references (13, 20, 24, 33). Furthermore, since ChatGPT 3.5′s training data cutoff is 2021, the treatment recommendations it provides may not be up to date.
(4) Data privacy and ethical issues. Most studies did not explicitly state whether HIPAA-compliant models were used, nor did they detail how patient data was anonymized; only a few studies explicitly indicated the use of compliant on-premises or proprietary models (27, 34).
(5) Methodological reporting is incomplete. According to an evaluation by Puce et al. using the METRICS checklist, most studies had incomplete reporting regarding prompt specificity, model transparency, and evaluation frameworks (29).

### 3.6 Assessment of the quality of reports included in the study

After excluding four review and opinion studies due to issues of applicability, this study assessed the reporting completeness of the 23 included studies using the MINIMAR framework, see Supplementary Appendix 2 for details (10). The overall MINIMAR compliance rate was 61.70%. Supplementary Appendix 2 presents the compliance rates for the four core components of MINIMAR: study population and setting, patient demographics, model architecture, and model evaluation. The compliance rates for each component were 100, 4.35, 78.26, and 61.96%, respectively. These findings indicate that current research on NLP/LLM in the field of sports injury assessment still has significant room for improvement in terms of reporting standards, and there is a need for more comprehensive reporting on demographic characteristics, data splitting, missing value handling, transparency, and external validation.

## 4 Discussion

This scoping review provides a comprehensive overview of the current applications of NLP and LLMs in the assessment of sports injuries and rehabilitation decision-making. The findings indicate that this field has experienced explosive growth since 2023, with research highly concentrated in North America, Europe, and East Asia, and primarily focused on algorithm validation. The vast majority of studies utilize simulated or synthetic question-answering data, while studies employing real clinical text are scarce and primarily concentrated on predictive tasks.

To date, no comprehensive study utilizing real clinical data has been reported. Of the 27 included studies, only six analyzed unstructured text derived directly from clinical practice; the remaining studies used questions formulated by researchers based on guidelines, question banks, or consensus documents. The application of LLMs to scenarios such as free-text clinical narratives, patient-written rehabilitation diaries, and multimodal clinical documents remains largely unvalidated. This imbalance directly impacts the generalizability of the research. Future studies should prioritize prospective evaluations using continuous real-world clinical data rather than question sets constructed retrospectively.

The included studies indicate that LLMs achieve moderate accuracy when answering sports injury-related questions—comparable to a 2nd-year orthopedic resident—and demonstrate moderate to good accuracy in clinical triage (11, 33). However, two major limitations persist. First, LLM performance depends heavily on prompt quality; the absence of detailed clinical information can substantially increase error rates. Second, LLMs lack genuine clinical reasoning capabilities and cannot currently perform inference grounded in pathophysiological mechanisms.

Although content generated by LLMs is structurally rigorous and factually accurate, its reading difficulty typically reaches a college level, far exceeding the sixth-grade level recommended by the American Medical Association (16, 20). This disparity means that populations with lower health literacy, who most urgently need clear and accessible guidance, may be effectively excluded from benefiting from these tools. Currently, LLMs lack adaptive simplification modes that tailor explanations to specific audiences, a gap that limits their equity as inclusive health communication tools.

The three types of “hallucinations” identified in this study—factual errors, guideline inconsistencies, and fictitious references—have different implications for clinical practice. Domain-specific AI platforms such as OpenEvidence substantially reduce guideline inconsistencies by grounding responses in authoritative sources (e.g., AAOS guidelines) and providing direct citation links to the underlying evidence. Retrieval-Augmented Generation (RAG) further mitigates fabricated content by anchoring model outputs to real-time, retrievable knowledge bases rather than relying exclusively on parametric memory. This suggests that for specific clinical tasks, training or fine-tuning domain-specific models may be more reliable than relying on general-purpose chatbots. However, OpenEvidence's interface and design are primarily geared toward healthcare professionals, making it difficult for the general patient population to use, which to some extent limits its direct application in patient education. In the future, a hybrid model could be explored that combines general-purpose LLMs, which provide preliminary explanations, with domain-specific models that provide evidence links.

The reliance on expert-only assessment, documented in the Results, has important methodological implications. Expert ratings of accuracy capture only one dimension of clinical utility; they do not reflect the patient- facing dimensions of comprehensibility, credibility, or emotional resonance that ultimately determine whether an LLM-generated recommendation translates into improved health behavior or outcomes. At the same time, an assessment of reporting quality based on the MINIMAR framework indicates that the completeness of reporting regarding patient demographics, data partitioning, handling of missing values, external validation, and optimization strategies is quite low. Reporting completeness for patient demographics stands at only 4.35%. This is not only an issue of reporting standards but also implies that existing studies struggle to evaluate the equity and potential biases of LLMs across different populations. The 4.35% patient demographic reporting compliance rate mirrors systemic deficits documented across the broader medical AI literature. In a systematic review of 390 clinical artificial intelligence and machine learning studies, Otokiti et al. (39) found that 84% of the models did not report the racial composition of their training data, 31% lacked gender data entirely, and only 16% used publicly available, non-proprietary datasets (39). A systematic review by Omar on demographic disparities in medical LLMs found that 91.7% of the studies (22 out of 24) identified measurable statistical biases, with 93.7% exhibiting gender bias and 90.9% exhibiting racial or ethnic bias. LLM-generated patient education materials exhibited statistically significant differences in reading grade level contingent on the implied racial context of the user (40). Given the ample evidence indicating significant disparities across racial, ethnic, and socioeconomic groups in the incidence of sports injuries, access to specialized medical care, and rehabilitation outcomes, the near-total absence of demographic reporting in the sports injury literature regarding NLP and LLMs is particularly concerning, as it renders even the most basic assessments of algorithmic fairness impossible (41). Looking ahead, journals and funding agencies should recommend that the recording of participant demographic data, pre-specified subgroup analyses, and algorithmic bias audits be made integral components of study design.

LLMs are currently evolving at an unprecedented pace, making comparisons with earlier models of limited relevance today. Recent analyses suggest that benchmark results for LLMs may remain valid for as little as 6–12 months; after that, published performance evaluations no longer reflect the actual capabilities of currently deployed systems. This rapid evolution poses unique challenges for evidence integration in AI literature reviews, as these changes may substantially alter performance between the time a paper is submitted and published (42). To mitigate this structural limitation, future research is advised to adopt the following measures: (a) precisely document model version identifiers, API endpoints, and access dates in all publications; (b) conduct routine re-benchmarking of key clinical tasks as new-generation models are released; and (c) develop benchmark datasets specific to sports medicine to enable reproducible longitudinal performance tracking across model generations (42). Until the relevant infrastructure is established, clinicians and researchers should be explicitly aware of the time-dependent nature of LLM performance data when interpreting it and should avoid extrapolating findings from outdated model versions to current clinical settings.

This highly concentrated geographic distribution stands in stark contrast to the global epidemiological patterns of sports injuries. In regions with high rates of sports injuries, such as South America, Africa, and Southeast Asia, relevant research is almost entirely absent. This may exacerbate the “algorithm gap” in global digital healthcare resources, meaning that the regions most in need of intelligent assistance are precisely those lacking locally validated models (6).

In terms of injury types, ACL injuries dominate, while other common injuries—such as Achilles tendon ruptures, rotator cuff injuries, and stress fractures—have not received sufficient attention. Furthermore, there is a marked lack of research covering the entire rehabilitation process, from acute-phase management to return-to-play decisions. The potential of LLMs in rehabilitation monitoring, adherence assessment, and psychological state analysis remains largely untapped. Sports injuries are highly sport-specific; evaluating the injury alone fails to fully account for the biomechanical characteristics and injury patterns of a specific sport. Future LLMs must shift from “general medical text processing” to context-based reasoning that deeply integrates “sport-specific biomechanical features.”

Current research has not addressed core scenarios in on-site team support, such as real-time injury assessment, training load monitoring, psychological status tracking, and return-to-play decision support. Nearly all existing studies rely on static, structured inputs rather than simulating how team physicians extract key information from colloquial, fragmented narratives. Prevention recommendations generated by LLMs suffer from redundancy and overgeneralization. To date, no study has evaluated the effectiveness of LLMs as “communication intermediaries” between athletes and team physicians, nor have the following three core capabilities identified been validated in an authentic on-site team medical environment: (1) Rapid, real-time speech-to-text transcription and key information extraction; (2) risk assessment that integrates the athlete's past injury history with subjective descriptions from the day of the competition; and (3) interpretability of output results and quantification of uncertainty.

Finally, issues of geographical and resource disparities are equally significant at the team physician level. In regions with scarce sports medicine resources (such as Africa and Southeast Asia), team physicians often lack specialized training. LLMs could theoretically serve as “remote knowledge assistants,” but existing models are trained on English-language and Western medical data, and differences in language, culture, and medical practices may lead to misjudgments. Therefore, future model deployment should support localized fine-tuning and multilingual, multicultural adaptation and establish a continuous feedback mechanism involving team physicians.

## 5 Limitations and future prospects

This study has the following limitations: the search was restricted to English-language literature, which may have resulted in the omission of important non-English studies; the exclusion of conference abstracts and preprints may have introduced publication bias; most of the included studies were cross-sectional algorithm evaluations, making a meta-analysis impossible; and no formal risk of bias assessment (e.g., PROBAST) was conducted for the included studies, which is a significant limitation. Finally, the rapid pace of LLM development means that the models evaluated in the included studies—predominantly GPT-3.5 and GPT-4—may no longer represent the current state of the art, limiting the temporal generalizability of the review's findings. Furthermore, as a scoping review, this study includes research with varying degrees of direct relevance to the core research question. Although we adopted a structured PCC framework, some of the studies covered areas such as general orthopedic knowledge assessment and multimodal electromyography analysis. While these areas are indirectly related to the assessment and rehabilitation of sports injuries, they nonetheless reflect the knowledge base and future directions in the field of sports injuries. Future updates to this review may consider adopting a more refined set of inclusion criteria to distinguish core applications.

Future research should focus on four interrelated core areas: grounded in sports settings, centered on athletes and team physicians, and focused on on-site team support scenarios; it should incorporate feedback from real athletes and their on-site medical teams to develop adaptive, simplified communication models tailored to different sports, health literacy levels, and competitive needs. Efforts should be made to enhance model reliability, expand coverage of injury types, annotate evidence levels and uncertainties, and reduce hallucinations and biases through domain-specific fine-tuning and retrieval-enhanced generation. To address geographical and research gaps, multi-center, localized deployments and multilingual adaptations should be implemented in regions with high rates of sports injuries but limited research. Research standards should be elevated by mandating the use of standardized reporting checklists such as TRIPOD-AI and MINIMAR, and by establishing public benchmark datasets and unified evaluation protocols (43, 44). Only by advancing these four directions simultaneously can we truly propel LLMs from “baseline evaluation” toward “clinical integration.”

## 6 Conclusion

NLP and LLMs have demonstrated potential as auxiliary tools in sports injury assessment and rehabilitation decision-making, capable of providing well-structured and accurate medical information. However, current research exhibits significant shortcomings in terms of readability, hallucination control, patient perspective, methodological reporting, and geographic coverage, which represent common limitations of current LLM applications in medicine. The transition from “baseline assessment” to “clinical integration” requires further prospective, multicenter, real-world studies, as well as the establishment of evaluation and reporting standards specifically tailored to sports medicine settings. Until then, LLMs should be positioned to “augment rather than replace” clinical decision-making, and their outputs must be reviewed and interpreted by healthcare professionals.

## Data Availability

The original contributions presented in the study are included in the article/supplementary material, further inquiries can be directed to the corresponding author.
