# Supplementary material for: Applications of natural language processing and large language models in sports injury assessment and rehabilitation decision-making: a scoping review
Source: Front Med (Lausanne). 2026 Jul 6;13:1866874. doi: 10.3389/fmed.2026.1866874 (PMC13382509; doi:10.3389/fmed.2026.1866874)
Supplement: Supplementary file 1 [file Table_1.docx]

**Appendix 1. Articles included in the review**

| **Author(s)(year)**  **Country** | **Research designs** | **Target population** | **Sports** | **Injuries and Clinical Conditions** | **NLP/LLM Methods** | **Text Data Sources** | **Specific NLP Tasks** |
| --- | --- | --- | --- | --- | --- | --- | --- |
| Rossettini et al(1)  2023  Italy | Viewpoint | Students, educators, clinicians, and researchers in the field of musculoskeletal rehabilitation | No specific sport | Comprehensive Sports Injury and Rehabilitation Management | ChatGPT (GPT-3.5, GPT-4), Microsoft Bing, Google Bard | Unstructured Text | Text generation, clinical reasoning support, information extraction and summarization, translation |
| Kaarre J et al(2) 2023 Finland | Algorithm Evaluation and Benchmarking \ Cross-sectional Studies | Patients, non-orthopedic healthcare providers | No specific sport | ACL Injury and Reconstruction Surgery | ChatGPT-4 | 20 frequently asked questions related to ACL surgery, identified through literature reviews, consensus statements, and common clinical inquiries | Medical question-answering systems, audience-adaptive text generation |
| Varady et al(3) 2024 United States | Algorithm Evaluation and Benchmarking \ Cross-sectional Studies | Patients, physicians | No specific sport | Medial Collateral Ligament Injury and Reconstruction Surgery | ChatGPT-4 | The most common patient questions (FAQs) and their corresponding answers, obtained from Google web searches and the ChatGPT-4 platform using the search term “ulnar collateral ligament reconstruction” | Medical question-answering systems, text generation and information extraction |
| Giorgino et al(4) 2024 Italy | Algorithm Evaluation and Benchmarking \ Cross-sectional Studies | Patients and their families, assessed by an orthopedic specialist | No specific sport | ACL Injury and Flatfoot | ChatGPT-3.5 and Google Bard | The 10 most common questions each extracted from the “People Also Ask” section of Google Search using the keywords “anterior cruciate ligament” and “flat feet” | Medical question-answering systems, text generation and patient education |
| Lum et al(5) 2024 United States | Algorithm Evaluation and Benchmarking | Orthopedic surgeons/orthopedic residents | No specific sport | Comprehensive Sports Injuries | ChatGPT-3.5 and Google Bard | 390 plain-text multiple-choice questions from the 2015–2016 and 2022 Orthopedic In-Training Examination (OITE) question banks | Medical exams and tests, medical question-answering systems, analysis of clinical knowledge recall and application |
| Kunze et al(6) 2024 United States | Algorithm Evaluation and Benchmarking \ Proof-of-concept analysis | Physicians | No specific sport | Knee Injuries | ChatGPT-4 | 20 simulated clinical scenarios constructed by researchers based on real-world clinical experience (including 10 brief chief complaints for triage and 10 expanded medical records containing more patient information) | Clinical triage, differential diagnosis generation, treatment and management plan generation |
| Quinn M et al(7) 2024 United States | Algorithm Evaluation and Benchmarking \ Cross-sectional Studies | Patients, assessed by an orthopedic sports medicine surgeon | No specific sport | ACL Injury and Reconstruction Surgery | ChatGPT-4, Gemini | Question prompts generated by adapting the 15 clinical practice guidelines (CPGs) on anterior cruciate ligament reconstruction published by the American Academy of Orthopaedic Surgeons (AAOS) in 2022 | Medical question-answering systems, patient education text generation |
| Naughton et al(8) 2024 Australia | Opinion | Sports scientists, physical therapists, team physicians | No specific sport | Comprehensive Assessment of Athlete Health and Performance, Emotional/Psychological Status, and Differential Diagnosis | ChatGPT, Microsoft's Copilot AI, Google's Bard/Gemini, VICTOR | Prompts for athlete oral and written texts, and medical imaging analysis | Question-answering systems and clinical recommendations, sentiment analysis |
| Saglam et al(9) 2025 Turkey | Algorithm Evaluation and Benchmarking \ Cross-sectional Study | Healthcare professionals in sports surgery and physical therapy | No specific sport | Comprehensive Sports Injuries | GPT-4 and GPT-3.5 | 10 standardized clinical scenarios constructed from real orthopedic clinical data extracted from hospital information systems over the past 10 years | Clinical decision support, diagnostic generation, treatment planning and rehabilitation plan generation |
| Rosen J et al(10) 2025 United States | Review | Physicians | No specific sport | Sports Injuries, Fractures | ChatGPT-4o, DeepSeek R1, and others | Unstructured data sources such as electronic health records, discharge summaries, consultation transcripts, and surgical records | Text Information Extraction (e.g., extracting data from surgical records), Text Content Generation (e.g., generating discharge documents and patient education materials), Diagnostic Support |
| Xing et al(11) 2025 China | Algorithm Evaluation and Benchmarking | Physicians | Functional movement screening | Comprehensive Sports Injuries | Large Language Model | Clinical testing | Information Extraction |
| Vivekanantha P et al(12) 2025 Canada | Algorithm Evaluation and Benchmarking \ Cross-sectional Study | Physicians | No specific sport | Patellar Instability and Medial Patellofemoral Ligament Reconstruction | ChatGPT-4o, Perplexity AI, Bing Copilot, Claude 2, and Google Gemini | 10 standardized clinical questions with high consensus selected from a previously published international Delphi Consensus study on patellar instability | Medical question-answering systems, Patient Education Information Generation |
| Liu et al(13) 2025 China/USA | Cross-sectional Analysis | Patients | No specific sport | Massive Irreparable Rotator Cuff Tears / Superior Capsular Reconstruction | Claude-3-Opus, GPT-4-Turbo, and Gemini-1.5-Pro | 11 common patient questions regarding capsular reconstruction surgery, jointly formulated by 10 clinical experts in sports medicine | Medical question-answering systems, Generating Preoperative Patient Education Materials/Informed Consent Documents |
| Puce L et al 2025(14) Italy | Systematic Literature Review | Athletes, recreational exercisers, patients with specific conditions, or the general population engaged in physical activity; physicians | No specific sport | Comprehensive Sports Injuries | ChatGPT-3.5, ChatGPT-4, Google Gemini | The UnoPerTutto metadata repository (integrating PubMed/MEDLINE, Scopus, and Web of Science), which ultimately included 10 relevant research articles | Text Generation (Exercise and Training Prescription Generation/Planning) |
| Diniz P et al(15) 2025 Luxembourg | Algorithm Evaluation and Benchmarking \ Retrospective Analysis | Physicians | Soccer | ACL Tears/Injuries | GPT-4o mini | Text from Search Result Items (SRIs) extracted using Google’s Programmable Search Engine (PSE) from selected multilingual news websites, club/football association official websites, and social media platforms (Instagram, Facebook, X/Twitter), based on injury records from the Transfermarkt.com database | Text translation, text relevance assessment, information extraction (identifying ACL tears, determining whether they are partial or complete, identifying related knee injuries, confirming surgical details) |
| Safran E et al(16) 2025 Turkey | Cross-sectional Study | Physical therapists, rehabilitation clinicians | No specific sport | Musculoskeletal Disorders and Rehabilitation | ChatGPT-4 | 20 specific questions developed by two experienced physical therapists specializing in musculoskeletal disorders, based on Clinical Practice Guidelines (CPGs) | Medical question-answering systems, text generation, clinical reasoning support, and rehabilitation plan generation |
| Zhang L et al 2025 China l(17) | Algorithm Evaluation and Benchmarking \ Cross-sectional Study | Physicians | No specific sport | Plantar Fasciitis | ChatGPT-4o and ChatGPT-4 Turbo | 21 clinical queries proposed in the 2023 American Physical Therapy Association (APTA) Clinical Practice Guideline on Plantar Fasciitis (including standard prompts and scenario-based prompts) | Medical question-answering systems, clinical guideline compliance assessment, and treatment recommendation generation |
| Zhu et al(18) 2025 China | Algorithm Evaluation/Framework Development and Validation | Athletes; physicians | No specific sport | Prevention and Treatment of Sports Injuries | Qwen2-0.5B-Instruct, GaLore, LoRA | The Chinese medical question-answering dataset cMedQA2, public literature and web resources, and high-quality sports injury question-answer pairs generated via human-in-the-loop feedback | Medical question-answering systems, text generation |
| Gültekin et al(19) 2025 Turkey | Algorithm Evaluation and Benchmarking \ Prospective Evaluation | Patients | No specific sport | ACL Injury | ChatGPT-4o/DeepSeek R1 | 10 frequently asked questions about ACL surgery from the webpages of sports medicine academic institutions | Medical question-answering systems, text generation, information extraction, and summarization |
| Wang S et al(20) 2026 China | Algorithm Evaluation and Framework Development | Patients; physicians | No specific sport | Sports Injuries and Rehabilitation | GPT-3, GPT-4, BERT, RoBERTa, XLNet | Two public review and rating datasets from Epinions and Amazon Sports & Outdoors (mapping product reviews to patient free-text feedback and ratings to tolerance scores) | Text encoding and sequence fusion, prediction of continuous and ordered tolerance scores, generation of structured exercise prescriptions and natural language explanations |
| Beda N et al(21) 2026 United States | Cross-sectional Study | Patients | No specific sport | Sports Injuries | ChatGPT 4.0 and Google Gemini | 40 standardized orthopedic patient questions (sourced from the “Frequently Asked Questions (FAQ)” section of Google Search and FAQs generated by large language models) | Medical question-answering systems, text generation, readability analysis of patient education texts |
| King B et al(22) 2026 United States | Cross-sectional Study | Patients | No specific sport | Sports Injuries | ChatGPT 4o, Gemini 2.5 Flash, and Grok 3 | 20 common patient questions regarding orthopedic biologic injections, jointly developed by a panel of experts | Medical question-answering systems, text generation, accuracy and readability assessment of patient education materials |
| Hsu W et al(23) 2026 Taiwan | Algorithm Evaluation and Benchmarking \ Cross-sectional Study | Orthopedic surgeons | No specific sport | Acute Isolated Meniscal Lesions | ChatGPT (GPT-4), Gemini, Claude (Claude 3.5 Sonnet), and OpenEvidence | 13 specific recommendations based on the 2024 American Academy of Orthopaedic Surgeons (AAOS) Clinical Practice Guideline (CPG) on acute isolated meniscal lesions (covering diagnosis, conservative treatment, and surgical intervention) | Medical question-answering systems, guideline compliance assessment, information reliability assessment |
| Miller et al(24) 2026 United States | Structured Case Study \ Retrospective Database Analysis and Algorithm Evaluation | Athletes, coaches, and primary care providers | Tennis, Squash, Badminton | Craniofacial Injuries | ChatGPT-4o | Ten years of retrospective injury data from the National Electronic Injury Surveillance System (NEISS), along with structured clinical vignettes developed by researchers based on data trends | Risk stratification, management recommendation generation, patient education material generation |
| Ko S et al(25) 2026 South Korea/USA | Algorithm Evaluation and Benchmarking | Physicians | No specific sport | Sports Injuries | Alibaba Qwen2.5-VL series (72B, 32B, 7B, 3B) and Meta Llama-3.2-Vision series (90B, 11B) | 210 questions from the 2023 Orthopedic In-Training Examination (OITE) (including 111 questions with images) | Multimodal medical question-answering and examination testing, clinical knowledge and reasoning assessment |
| Halvorson R et al(26) 2026 United States | Retrospective Cross-sectional Study | Physicians | No specific sport | Knee and Shoulder Injuries | ChatGPT-4o | Previsit questionnaire responses containing patient chief complaints, symptoms, and treatment history, as well as radiological MRI report text | Clinical triage decision support, text classification and prediction (predicting the need for advanced imaging studies and surgery based on unstructured text) |
| Bandara et al(27) 2026 United States | Algorithm Evaluation \ Framework Development and Validation | Physicians | No specific sport | Neuromuscular Status Assessment | Fine-tuned Llama-Vision, Pixtral-Vision, Qwen2-VL, gpt-oss | EMG waveform images and associated text datasets including expert clinical observations, recovery timelines, and athlete metadata annotations | Multimodal analysis, text generation |

Table2 Key Findings and Limitations from the Original Text

| **Author(s)(year)**  **Country** | **Core Findings** | **Limitations** |
| --- | --- | --- |
| Rossettini et al(1)  2023  Italy | In this Viewpoint, we describe the potential applications and limitations, and recommended actions for education, clinical practice and research when using AI chatbots for musculoskeletal rehabilitation management, aspects that may have similar implications for the broader health care community | Chatbots increase the risk of plagiarism and copyright disputes over the material copied from other documents. They may provide inaccurate sources/references and may produce credible - albeit - incorrect answers. Chatbots can also 'hallucinate'-inventing terms when information is limited-generating superficial and misleading answers ... Chatbots can generate repetitive and redundant text, lacking in creativity, originality, and analysis when deliberating complex health care concepts. ...having been trained by scraping data up to 2021, have restricted knowledge, potentially limiting content generation at risk for dated recommendations. |
| Kaarre J et al(2) 2023 Finland | Overall, ChatGPT was successful in generating correct responses in approximately 65% of the cases related to ACL surgery. The findings of this study imply that LLMs offer potential as a supplementary tool for acquiring orthopaedic knowledge. However, although ChatGPT can provide guidance and effectively adapt to diverse target audiences, it cannot supplant the expertise of orthopaedic sports medicine surgeons in diagnostic and treatment planning endeavours due to its limited understanding of orthopaedic domains and its potential for erroneous responses. | This study has several limitations. The reliability of the responses generated by ChatGPT was not evaluated, inviting the possibility that responses may have differed if the same question had been asked repeatedly, or if the responses had been ordered differently. Furthermore, ChatGPT-4 as of March 14th, 2023, was used, which is only one type of LLM. Future studies should consider evaluating multiple LLMs to prove a more comprehensive assessment. The three-point response scale used to evaluate responses was not standardized and, therefore, may have limited the objective measurement of correctness, completeness, and adaptability. Thus, the different assessors may have interpreted the scale differently, leading to inconsistencies in the assessment process. To try to mitigate this threat, the same instructions were provided to all assessors and included examples of how to use the scales. Moreover, the four orthopaedic sports medicine surgeons who assessed the responses were not blinded to the fact that the responses were generated by ChatGPT. Therefore, the assessment of the reviewers may have been influenced both by individual bias and their preconceptions about the correctness of LLMs. |
| Varady et al(3) 2024 United States | ChatGPT-4 is capable of providing responses with clinically relevant content concerning UCL injuries and reconstruction. ChatGPT-4 utilized a greater proportion of academic websites to provide responses to FAQs representative of patient inquiries compared with Google Web Search and provided significantly more accurate answers. Moving forward, ChatGPT has the potential to be used as a clinical adjunct when answering queries about UCL injuries and reconstruction, but further validation is warranted before integrated or autonomous use in clinical settings. | First, both Google and ChatGPT-4 are dynamic resources that evolve over time. As such, the current results may not always hold in the future. Second, the study is limited to UCL injuries and reconstruction, and the results may not be generalizable to other conditions. ... Third, the performance of a limited number of questions was assessed, which does not represent all possible questions or concerns a patient may have about this topic. ... Fourth, although some statistically significant differences were observed between ChatGPT-4 and Google ... it is unclear whether these differences are clinically significant. Fifth, patients may combine information from multiple resources (ie, both Google and ChatGPT-4), which was not assessed in this study. Last, although a methodological strength of this study is utilizing a new browser and erasing history with each iterative search, it is also a limitation because it may ultimately affect the generalizability of results as others may not achieve similar results if they do not refresh their browsers. |
| Giorgino et al(4) 2024 Italy | ChatGPT-3.5 and Google Bard yielded good-quality responses, with average scores of 4.1 ± 0.7 and 4 ± 0.78, respectively, for sports medicine. For pediatric orthopedics, Google Bard scored 3.5 ± 1, while the average score for responses generated by ChatGPT was 3.8 ± 0.83. In both cases, no statistically significant difference was found between the platforms (p = 0.6787, p = 0.3092). Despite ChatGPT's responses being considered more readable, both platforms showed promise for AI-driven patient education, with no reported misinformation. | Firstly, the decision to restrict the analysis to ten questions may represent a substantial intrinsic limitation, as it only covers part of the spectrum of possible topics or issues patients may have in the field of sports medicine. ... Furthermore, as mentioned in the document, the limited training period of ChatGPT may affect its ability to provide updated and relevant information, especially considering the continuously evolving nature of medical knowledge. Another limiting aspect is the absence of an analysis based on patient feedback. Patient perceptions and needs may differ from expert evaluations, and the omission of this perspective could reduce the completeness of the paper's conclusions regarding practical utility for individuals seeking medical information. |
| Lum et al(5) 2024 United States | BARD answered more overall questions correctly (58% vs 54%, p<0.001). ChatGPT performed better in sports medicine and basic science and worse in hand surgery, while BARD performed better in basic science (p<0.05). The AIs performed better in recall questions compared to the application of knowledge (p<0.05). Based on previous data, it ranked in the 42nd-96th percentile for post-graduate year ones (PGY1s), 27th-58th for PGY2s, 3rd-29th for PGY3s, 1st-21st for PGY4s, and 1st-17th for PGY5s... BARD performed better than ChatGPT overall. Although the AI reached the second-year PGY orthopedic resident level, it fell short of passing the American Board of Orthopedic Surgery (ABOS). | This study has several limitations, particularly the inability to incorporate visual identification, interpretation, and integration within the questions. Almost half of the questions contained images, figures, or charts, leading to their exclusion. ... The exclusion of image-based questions may have biased the results by potentially omitting more challenging or application-focused questions for the LLM. Moreover, the basic science sub-specialty contained more recall-based questions, which could have inflated the LLM's performance in that area. ... General limitations of AI models include potential biases or inaccuracies in the datasets they are trained on, which can reflect or amplify existing societal biases or inequalities and may contain outdated information. Lastly, limitations specific to this LLM stem from its training on broad, non-specific information. While it excels in summarization, translation, and text generation, it might struggle with context or nuanced language in specialized knowledge areas, leading to inaccurate or misleading responses. |
| Kunze et al(6) 2024 United States | All ChatGPT-4 diagnoses were deemed appropriate within the spectrum of potential pathologies on a differential. The top diagnosis on the differential was identical between surgeons and ChatGPT-4 for 70% of scenarios, and the top diagnosis provided by the surgeon appeared as either the first or second diagnosis in 90% of scenarios. Overall, 16 of 30 diagnoses (53.3%) in the differential were identical. When provided with 10 expanded vignettes with a single diagnosis, the accuracy of ChatGPT-4 increased to 100%, with the suitability of management graded as appropriate in 90% of cases. Specific information pertaining to conservative management, surgical approaches, and related treatments was appropriate and accurate in 100% of cases. | First, a limited number of triage and clinical vignettes were provided. Second, this search was performed in a single geographic region of the United States. Third, grading of the responses provided by ChatGPT-4 was inherently subjective. Fourth, inputs were given to ChatGPT-4 at a single time point which reflect training data curated by time-sensitive data availability as well as supervised and reinforcement training from humans. Fifth, acuity of triage was not systematically assed for in the current study, which may be relevant depending on the clinical use of ChatGPT-4 for diagnosing certain knee conditions. Sixth, prompts were not input multiple times. Finally, it is possible that the specific wording of prompts may influence, predisposing to prompt engineering bias |
| Quinn M et al(7) 2024 United States | Overall, both LLMs performed well with mean scores >4 for the 5 key characteristics. Gemini demonstrated superior performance in overall clarity (4.848 ± 0.36 vs 4.743 ± 0.481, P = .034), but all other characteristics demonstrated nonsignificant differences (P > .05). Gemini also demonstrated superior clarity in the surgical timing and technique (P = .038) as well as the prevention and rehabilitation (P = .044) subcategories. Additionally, Gemini had superior performance completeness scores in the rehabilitation and prevention subcategory (P = .044), but no statistically significant differences were found amongst the other subcategories. The overall IRR was found to be 0.71 (moderate). | This study is not without limitations. Given that this is a novel area of research, we were unable to calculate a power analysis to ensure an appropriate sample size. We also translated the AAOS clinical practice guidelines into questions to prompt ChatGPT-4 and Gemini, responses which may have introduced bias into our study. Additionally, given the rapid evolution of this technology, it is important to note that more advanced versions of these AI platforms are available with ChatGPT-4o and Gemini Advanced. However, to maximize the generalizability of our results, the free versions of these platforms were used for response generation because the most capable versions of these platforms require a subscription and therefore may not be accessible to all patients. Furthermore, surgeon bias may have influenced response grading; however, we attempted to control for this by blinding the surgeons to the source of each response. Certain investigation categories such as clarity are particularly subjective and may even be better judged by patients themselves. Finally, although the grading system used to rate responses was based on previously published studies, it has not been formally validated and therefore limits the interpretability of these results. |
| Naughton et al(8) 2024 Australia | The automation and AI boom also brings substantial opportunities. Among them are automated sentiment analysis and Digital Twin technologies which may reveal novel insights into athlete health and wellbeing and team tactical patterns, respectively. However, without due consideration of the interactions between humans and technology in the broader system of sport, adverse impacts are likely to be felt. Human and AI teamwork may require new ways of thinking. | Paradoxically, replacing humans with technology increases system complexity and makes them more prone to failure. Other potential issues which have yet to be adequately rectified include a high risk of bias, data governance and other ethical issues (e.g., prevention of harm, fairness, privacy, transparency and explainability, accountability etc.), malicious use (e.g., development of new performance enhancing drugs), and a lack of established performance, regulation, and safety in real-world settings where teams of workers and AI work co-operatively. |
| Saglam et al(9) 2025 Turkey | GPT-4 significantly outperformed GPT-3.5 across all evaluated criteria. Paired t-test results (t(55) = 10.45, p < 0.001) demonstrated that GPT-4 provided more accurate diagnoses, superior treatment plans, and more detailed surgical recommendations. ANOVA results confirmed the higher suitability of GPT-4 in treatment planning (F(1, 55) = 35.22, p < 0.001) and rehabilitation protocols (F(1, 55) = 32.10, p < 0.001). Cronbach's alpha values indicated higher internal consistency for GPT-4 (α = 0.478) compared to GPT-3.5 (α = 0.234), reflecting more reliable performance. | First, the study was limited to GPT-4 and GPT-3.5, meaning that the findings may not be generalizable to other AI models such as DeepSeek, Llama, or Gemini. ... Additionally, the study relied on standardized clinical scenarios, which, while designed to reflect real-world cases, may not fully capture the variability and complexity of actual patient cases. ... Another limitation is the potential bias in scenario creation, as two orthopedic surgeons and two physiotherapists reviewed and finalized the cases. ... Finally, AI models, including GPT-4, are susceptible to "hallucination" (the generation of inaccurate or misleading information). |
| Rosen J et al(10) 2025 United States | Each algorithm offers strengths in accuracy, efficiency or scalability, but also carries bias, transparency, computational cost and lack of external validation. This review explores how these algorithms are shaping orthopaedics, highlighting their benefits, limitations and challenges. Rigorous validation, transparent reporting and governance are essential for safe clinical use. | This review has several limitations. This review was restricted to English-language studies published between 2021 and 2025. While selected seminal works published before this period were included to provide historical and methodological context, it remains possible that other relevant earlier studies were not captured. Language or publication bias may also have been introduced. Formal bias assessment tools were not used which limits evaluation of study quality. The rapidly evolving nature of AI means that some findings may become outdated. Finally, the search was limited to two databases, so additional relevant studies may not have been captured. |
| Xing et al(11) 2025 China | demonstrate that our approach significantly outperforms existing methods while offering superior interpretability. Experimental results demonstrate that our approach outperforms existing methods in terms of accuracy and interpretability, with a substantial increase in the clarity and detail of the rationales provided. These findings highlight the potential of our framework for fine-grained action quality assessment with the aid of LLMs. © 2025 Xing et al. This is an open access article distributed under the terms of the Creative Commons Attribution License, which permits unrestricted use, distribution, and reproduction in any medium, provided the original author and source are credited. | prevents the effective application of automated FMS evaluation for injury prevention and rehabilitation. We develop a fine-grained, hierarchical FMS dataset, LLM-FMS, derived from FMS videos and enriched with detailed, hierarchical action annotations. This dataset comprises 1812 action keyframe images from 45 subjects, encompassing 15 action representations of seven FMS actions. Each action includes a score, scoring criteria, and weight data for body parts. To our extensive knowledge, LLM-FMS is the first fine-grained fitness action dataset for action evaluation task. Additionally, a novel framework for action quality assessment based on large language models (LLMs) is proposed, designed to enhance the interpretability of FMS evaluations. Our method integrates expert rules, utilizes RTMPose to extract key skeletal-level action features from key frames, and inputs prompts into the LLM, enabling it to infer scores and provide detailed rationales. Experimental results demonstrate that our approach significantly outperforms existing methods while offering superior interpretability. Experimental results demonstrate that our approach outperforms existing methods in terms of accuracy and interpretability, with a substantial increase in the clarity and detail of the rationales provided. These findings highlight the potential of our framework for fine-grained action quality assessment with the aid of LLMs. © 2025 Xing et al. This is an open access article distributed under the terms of the Creative Commons Attribution License, which permits unrestricted use, distribution, and reproduction in any medium, provided the original author and source are credited. |
| Vivekanantha P et al(12) 2025 Canada | ChatGPT4o and Claude2 had the highest percentage of reviews (38/80, 47.5%) considered to be an “excellent response not requiring classification”, or a Mika score of 1. Google Gemini had the highest percentage of reviews (17/80, 21.3%) considered to be “unsatisfactory requiring substantial clarification”, or a Mika score of 4 (p < 0.001). The median ± interquartile range (IQR) Mika scores was 2 (1) for ChatGPT4o and Perplexity AI, 2 (2) for Bing CoPilot and Claude2, and 3 (2) for Google Gemini. Median responses were not significantly different between ChatGPT4o, Perplexity AI, Bing CoPilot, and Claude2, however all four statistically outperformed Google Gemini (p < 0.05). Inter-rater agreement was classified as moderate (0.40 > AC2 ≥ 0.60) for ChatGPT, Perplexity AI, Bing CoPilot, and Claude2, while there was no agreement for Google Gemini (AC2 < 0). | First, surgeons were not blinded when reviewing responses from different LLMs, which may introduce bias. Second, despite moderate agreement as per Gwet's AC2 coefficient for most LLMs, having humans assess the accuracy of responses introduces a level of bias within grading. Third, LLMs are updated frequently, and the quality of responses are likely to improve with each subsequent update. Finally, this study only evaluated 10 questions related to PFI, limiting the ability to assess a broader and more diverse range of issues across this field. Capturing this phenomenon is not possible with a cross-sectional design. |
| Liu et al(13) 2025 China/USA | For the 11 query responses, clinicians rated Gemini significantly higher than Claude in all categories (P<.05) and higher than GPT in completeness, risk avoidance, and overall rating (P<.05). For the 3 educational documents, Gemini's Patient Education Materials Assessment Tool score significantly exceeded Claude's (P=.03), and patients rated Gemini's materials superior in all aspects, with significant differences in educational quality versus Claude (P=.02) and overall satisfaction versus both Claude (P<.01) and GPT (P=.01). GPT had significantly higher readability than Claude on 3 R-based metrics (P<.01). Interrater agreement was high among clinicians and fair among patients. Conclusions: Claude-3-Opus, GPT-4-Turbo, and Gemini-1.5-Pro effectively generated readable presurgical education materials but lacked citations and failed to discuss alternative treatments or the risks of forgoing SCR surgery, highlighting the need for expert oversight when using these LLMs in patient education. | This study has several limitations. First, both the linguistic input and the analyzed responses were in Chinese. On one hand, this choice was made to facilitate assessments by Chinese-speaking clinical experts and patients during follow-ups. On the other hand, input in different languages could introduce potential errors and biases. Second, this research only explores the feasibility of using LLMs to generate content related to SCR for patient education. The variability in surgical procedures and specialties could pose distinct challenges in patient education, which means the conclusions drawn from this study cannot be simply generalized to other disciplines. Finally, during the "Prompts Development" phase, it was found that without additional background information, SCRs are prone to be misidentified by LLMs as bridge suture repairs of the supraspinatus muscle. However, since all 3 models used were proprietary, we opted for a "Background+ Question" approach to mitigate this systematic error, without being able to investigate the reasons behind such occurrences. |
| Puce L et al 2025(14) Italy | Findings indicate that generative AI-generated training programs generally adhere to established exercise guidelines but often lack specificity, progression, and adaptability to real-time physiological feedback. AI-generated recommendations were found to emphasize safety and broad applicability, making them useful for general fitness guidance but less effective for high-performance training. GPT-4 demonstrated superior performance in generating structured resistance training programs compared to older AI models, yet limitations in individualization and contextual adaptation persisted. | The findings of the studies included in this systematic review are limited by the small sample sizes in many studies often with few or no real participants which restricts the generalizability of the results. |
| Diniz P et al(15) 2025 Luxembourg | Verification of 231 athletes yielded 1546 SRIs. Human analysis of the SRIs showed that 335 mentioned an ACL tear, corresponding to 83 athletes with ACL tears. Specificity and sensitivity of GPT in identifying mentions of ACL tears in a player were 99.3% and 88.4%, respectively. ... Conclusion: This study shows that an AI-powered framework can achieve high specificity in cross-checking ACL tear reports in male professional football from public databases, markedly reducing manual workload and enhancing the reliability of media-based sports medicine research. | The primary limitation of this study is that, despite our cross-checking procedure, we could not definitively confirm diagnoses. While prior research has deemed the press a reliable source for severe injuries like ACL tears [19], it cannot replace the accuracy of patient clinical records... Additionally, our study concentrated exclusively on ACL tears in male professional football players, limiting the generalisability of the results to other injuries or sports. Finally, the AI-powered framework leads to fewer athletes being included in the study than using Transfermarkt data directly, as third-party confirmation is not always available—a necessary compromise to ensure data reliability. |
| Safran E et al(16) 2025 Turkey | This study demonstrates ChatGPT's potential in providing guideline-aligned information in musculoskeletal rehabilitation. However, due to observed limitations in consistency, completeness, and the ability to replicate nuanced clinical reasoning, its use should remain supplementary rather than as a primary decision-making tool. While it performed better in disease information, as evidenced by higher inter-rater agreement and scores, its performance in the rehabilitation category was comparatively lower, highlighting challenges in addressing complex, nuanced therapeutic interventions. This variability in consistency and domain-specific reasoning underscores the need for further refinement to ensure reliability in complex clinical scenarios. | This study has several limitations that should be acknowledged. First, only two assessors were involved in the evaluation, which may limit the generalizability of inter-rater agreement results. Second, the assessors also developed the questions, introducing potential bias despite independent scoring and statistical analysis of agreement. Third, while the assessment criteria were informed by existing literature and expert consensus, the rating tool itself has not been psychometrically validated. Additionally, the study was limited to a fixed set of 20 questions, which, although diverse, may not fully represent the range of real-world clinical scenarios. Finally, the findings reflect ChatGPT's performance during a specific time window and may not apply to future updates of the model. |
| Zhang L et al 2025 China l(17) | On the dimensions of accuracy, consistency, self-awareness, and fabrication and falsification, the two mainstream versions of ChatGPT showed equivalent superior performance in the generation of recommendations concordant with clinical guidelines for the management of PF. However, specific issues including performance variations between different prompt strategy, recommendation grade, and recommendation type should be noted, and the models should still be utilized with caution. | This study has several limitations. First, this study utilized ChatGPT only and did not evaluate other prevailing LLMs... Second, ChatGPT models undergo continuous updates, which could affect the reproducibility of our results... Third, while this study developed a scenario-based prompting approach to simulate real-world patient inquiries, this methodology cannot entirely encompass the complexity and variability of actual patient interactions. Fourth, only 21 queries were formulated based on the recommendations of a single CPG... |
| Zhu et al(18) 2025 China | Experiment results show that the proposed method enhanced by GaLore is superior to SOTA methods such as low-rank adaptation (LoRA) in terms of convergence accuracy, training time, memory consumption, and indicators of BLEU-4 and ROUGE-2. Meanwhile, the empirical effect of injury prevention Q&A cases indicate that Qwen2-0.5B-Instruct trained by the proposed method have obvious advantages in professional knowledge understanding and overcoming hallucinations. | During the experiments, it was found that when the rank of GaLore was 1,024, the evaluation metrics were lower than those with a rank of 512. ... indicating that LoRA is not as effective under the current settings and requires further optimization and adjustment. Future work will focus on enhancing the retrieval-augmented generation capabilities of LLMs in combination with knowledge graphs. |
| Gültekin et al(19) 2025 Turkey | Both models demonstrated high accuracy (mean scores of 3.9/4) and consistency (4/4). Significant differences were observed in clarity and completeness: ChatGPT provided more comprehensive responses (mean completeness 4.0 vs. 3.2, p < 0.001), while DeepSeek's answers were clearer and more accessible to laypersons (mean clarity 3.9 vs. 3.0, p < 0.001). DeepSeek had lower FKGL (8.9 vs. 14.2, p < 0.001) and higher FRES (61.3 vs. 32.7, p < 0.001), indicating greater ease of reading for a general audience. | This study is not without limitations. The sample consisted of only ten questions, selected to reflect common patient inquiries, but was insufficient to capture the full variability in model performance. These questions were general in nature—focusing on topics such as injury mechanisms and rehabilitation—and did not evaluate the AI's ability to address more complex clinical scenarios, which may present additional challenges in clarity and accuracy. Additionally, evaluations were conducted by medical professionals rather than patients, which may limit the generalizability of readability and clarity assessments. |
| Wang S et al(20) 2026 China | The experimental results demonstrate that integrating large-scale language understanding with task-specific sequence modelling and adaptive text-rating alignment yields a robust, data-efficient foundation for intelligent clinical support in personalised sport-training rehabilitation. | Limitations. First, the current study relies on proxy datasets lacking ground-truth physiological signals; integrating real-world wearables will further validate clinical applicability. Second, while ReLite yields interpretable rationales via attention heat-maps and LLM prompts, formal user studies with physiotherapists are needed to quantify trust and adoption barriers. The current validation is based on Epinions and Amazon Sports datasets, which serve as proxies for real rehabilitation logs. ... The generated exercise prescriptions and natural language justifications have not been systematically evaluated by licensed physiotherapists or clinicians. |
| Beda N et al(21) 2026 United States | LLM responses were significantly longer and more complex than Google Search snippets. Average response lengths were 342.75 words for ChatGPT 4.0, 306.88 words for Google Gemini, and 40.18 words for Google Search (p < 0.001). FRE scores indicated difficult readability for ChatGPT (25.6) and Gemini (26.0) versus a significantly easier comprehensibility for Google Search (40.7, p < 0.05). FKGL analysis showed ChatGPT responses required a higher reading level (13.7) than Google Search (12.6, p < 0.05). Source analysis of Google Search revealed that 55% of results were from academic sites, 32.5% from medical practices, 7.5% from single surgeons, 2.5% from government websites, and 2.5% from social media, with no commercial websites represented. LLMs did not provide explicit source citations | This study focused on response length, readability, and source characterization, but did not evaluate the factual accuracy of responses, as prior work has already addressed variability in LLM medical correctness. In addition, only initial, single-prompt responses were analyzed; incorporating follow-up prompts may yield different results and should be explored in future work. Finally, Google Search snippets were analyzed in isolation without considering linked webpages, which may provide additional depth and nuance. Together, these considerations highlight areas for future research, including accuracy assessment, multi-turn interactions, and expansion to a wider range of orthopedic and patient-centered topics. |
| King B et al(22) 2026 United States | Interrater reliability was strong for all accuracy ratings (ICCs >0.70; P < .05). While response accuracy was generally acceptable, 50% (10/20) of ChatGPT, 25% (5/20) of Gemini, and 30% (6/20) of Grok responses were deemed as requiring more than minimal clarification (CRRS >2). One-way matched analysis of variance (ANOVA) revealed a significant effect of AI model on both CRRS (P = .02) and AIRM scores (P = .02), with Gemini displaying improved accuracy compared with ChatGPT (CRRS, P = .04; AIRM, P = .03). Regarding readability, the mean FKGL of all 3 models was at a collegiate level or higher, and all responses exceeded the American Medical Association and National Institutes of Health-recommended 6th-grade reading level for patient education. One-way matched ANOVA revealed a significant effect of AI model on FKGL (P = .02), with Gemini displaying reduced readability compared with ChatGPT (P = .03) | However, this study has several limitations. First, responses were captured at a single time point, and given the rapid evolution of AI models, outputs from future versions of AI LLMs may differ substantially. Additionally, because the orthobiologic evidence base remains heterogeneous, accuracy assessments reflect the interpretation of a limited number of expert reviewers rather than comparison against settled scientific truth. The expert raters were also the same individuals who generated the question list, which may have introduced bias in the accuracy ratings. Second, only 20 questions were analyzed, which, while representative of common clinical inquiries, cannot capture the entire spectrum of orthobiologic concerns. Third, readability was assessed using a single validated tool (FKGL), which may not fully represent real-world comprehension. Finally, while physician raters ensured alignment with medical evidence, patient perspectives were not incorporated... |
| Hsu W et al(23) 2026 Taiwan | OpenEvidence demonstrated the highest concordance with the 2024 AAOS guidelines (100% appropriate), followed by ChatGPT (84.6%), Gemini (84.6%), and Claude (76.9%). OpenEvidence also achieved the highest JAMA score (mean 3.69), reflecting its superior reliability and provision of specific citations compared to the other LLMs. However, overall readability for all LLMs remained at a high-school to collegiate level, exceeding the recommended literacy level for general patient education. | First, the study was conducted at a single time point; as LLMs are constantly updated, their performance may evolve. Second, the evaluation was based on a specific set of 13 questions derived from one CPG, which may not encompass all facets of meniscal pathology management. Third, while OpenEvidence performed exceptionally well, it is a subscription-based, domain-specific model, which may limit its accessibility to all patients compared to free versions of other LLMs. |
| Miller et al(24) 2026 United States | NEISS data revealed that squash carries the highest risk of craniofacial injury per player, particularly for ocular and orbital fractures. ChatGPT was able to successfully stratify risk and provide sport-specific management and treatment advice based on structured injury vignettes. The model demonstrated potential in translating complex injury data into actionable safety recommendations for coaches and athletes. However, the readability of AI-generated content still requires optimization to be suitable for all levels of athletes and families. | The primary limitation is the retrospective nature of the NEISS database, which may underreport injuries not treated in emergency departments. For the AI portion, ChatGPT's performance was evaluated based on simulated vignettes rather than real-time clinical encounters. Additionally, the risk of "hallucinations" in medical advice remains a concern, and the generated content was not yet validated for prospective injury reduction in a field setting. |
| Ko S et al(25) 2026 South Korea/USA | The 2 largest open-source models, Qwen2.5-VL-72B and Llama-3.2-90B, demonstrated performance levels comparable to those of second-year orthopedic residents on the OITE examination. A mid-sized model, Qwen-32B, slightly outscored first-year residents. In contrast, small-sized models (under 11 billion parameters) performed worse than first-year residents. Qwen2.5-VL-72B performed best in foot & ankle and sports medicine topics, while Llama-3.2-90B was strongest in basic science and hand & wrist. All models had the most difficulty with spine and pediatric questions. Overall, model accuracy increased steadily with model size up to 72 billion parameters, but larger sizes showed little additional improvement. | This study has several limitations. First, the generalizability of our findings may be limited by reliance on a single test (the 2023 OITE). Performance on a multiple-choice written exam with selected images may not fully reflect a model's ability to address the complexity of real-world clinical scenarios. Second, there is a possibility that models were exposed to OITE questions during training, raising concerns about memorization versus genuine reasoning. Third, frequent model updates can lead to performance shifts over time. Our findings reflect a performance snapshot based on the specific model versions evaluated. Fourth, we did not examine the impact of temperature settings. Prior work suggests that increasing temperature may improve diagnostic accuracy in medical imaging tasks, 24) though at the cost of reduced response consistency. We did not assess the quality of the models' explanatory outputs, which may influence clinical utility. |
| Halvorson R et al(26) 2026 United States | In the combined cohort of 1141 patients, the LLM predicted surgeon recommendation for advanced imaging with 70% accuracy, 83% sensitivity, and 64% specificity using previsit questionnaire responses alone. Imaging predictions were accurate for common diagnoses, including anterior cruciate ligament (ACL, 94%), meniscus (85%), and rotator cuff (80%) injuries but poor for knee (54%) and shoulder arthritis (66%). When augmented with imaging reports, the LLM predicted recommendations for surgery with 81% accuracy, 88% sensitivity, and 72% specificity. Surgical predictions were highly accurate for ACL (93%), meniscus (78%), rotator cuff (83%), and shoulder instability related pathologies (78%). | First, surgeon recommendations, which themselves are prone to bias, errors, or between-provider differences, were referenced as the gold standard for patient management. The data collected for this study originate from a single institution; further validation on prospective, and external data is necessary for future validation. ... Given that the models in this analysis use only unstructured free-text as input, traditional feature attrition analyses could not be performed. Thus, it is difficult to understand the relative weighting placed on specific input data (e.g., specific questions or MRI findings) by the LLM in making classifications. Another limitation is the possibility for selection bias in the imaging-augmented surgical decision model... Finally, our model relies on pretrained models not specifically tailored to orthopaedic surgery. |
| Bandara et al(27) 2026 United States | Experimental evaluation demonstrated that the proposed hybrid system delivers accurate, consistent, and clinically interpretable assessments of neuromuscular states, including fatigue, injury, and recovery, directly from EMG waveform images and contextual metadata. Compared with baseline models, the fine-tuned VLM consortium exhibited substantially improved precision, consistency, and contextual awareness, while the reasoning LLM enhanced diagnostic coherence through cross-model consensus and structured reasoning, thereby supporting responsible and explainable AI-driven decision making. | While the proposed platform demonstrates strong performance in automated H-reflex interpretation, it is designed to complement rather than replace existing clinical and signal-processing workflows. Traditional electromyographic analysis and clinician-supervised interpretation remain the gold standard in diagnosis. ... Broader adoption and possible transition to partial automation will require prospective validation in diverse patient populations, parameter-sensitivity benchmarking, and alignment with regulatory and ethical standards for medical decision-support systems. |

**References**

1. Rossettini G, Cook C, Palese A, Pillastrini P, Turolla A. Pros and Cons of Using Artificial Intelligence Chatbots for Musculoskeletal Rehabilitation Management. *Journal of Orthopaedic & Sports Physical Therapy* (2023) 53:728–734. doi: 10.2519/jospt.2023.12000

2. Kaarre J, Feldt R, Keeling LE, Dadoo S, Zsidai B, Hughes JD, Samuelsson K, Musahl V. Exploring the potential of ChatGPT as a supplementary tool for providing orthopaedic information. *Knee surg sports traumatol arthrosc* (2023) 31:5190–5198. doi: 10.1007/s00167-023-07529-2

3. Varady NH, Lu AZ, Mazzucco M, Dines JS, Altchek DW, Williams RJ, Kunze KN. Understanding How ChatGPT May Become a Clinical Administrative Tool Through an Investigation on the Ability to Answer Common Patient Questions Concerning Ulnar Collateral Ligament Injuries. *Orthopaedic Journal of Sports Medicine* (2024) 12:23259671241257516. doi: 10.1177/23259671241257516

4. Giorgino R, Alessandri-Bonetti M, Del Re M, Verdoni F, Peretti GM, Mangiavini L. Google Bard and ChatGPT in Orthopedics: Which Is the Better Doctor in Sports Medicine and Pediatric Orthopedics? The Role of AI in Patient Education. *Diagnostics* (2024) 14:1253. doi: 10.3390/diagnostics14121253

5. Lum ZC, Collins DP, Dennison S, Guntupalli L, Choudhary S, Saiz AM, Randall RL. Generative Artificial Intelligence Performs at a Second-Year Orthopedic Resident Level. *Cureus* (2024) 16:e56104. doi: 10.7759/cureus.56104

6. Kunze KN, Varady NH, Mazzucco M, Lu AZ, Chahla J, Martin RK, Ranawat AS, Pearle AD, Williams RJ. The Large Language Model ChatGPT-4 Exhibits Excellent Triage Capabilities and Diagnostic Performance for Patients Presenting With Various Causes of Knee Pain. *Arthroscopy* (2025) 41:1438-1447.e14. doi: 10.1016/j.arthro.2024.06.021

7. Quinn M, Milner JD, Schmitt P, Morrissey P, Lemme N, Marcaccio S, DeFroda S, Tabaddor R, Owens BD. Artificial Intelligence Large Language Models Address Anterior Cruciate Ligament Reconstruction: Superior Clarity and Completeness by Gemini Compared With ChatGPT-4 in Response to American Academy of Orthopaedic Surgeons Clinical Practice Guidelines. *Arthroscopy* (2025) 41:2002–2008. doi: 10.1016/j.arthro.2024.09.020

8. Naughton M, Salmon PM, Compton HR, McLean S. Challenges and opportunities of artificial intelligence implementation within sports science and sports medicine teams. *Front Sports Act Living* (2024) 6:1332427. doi: 10.3389/fspor.2024.1332427

9. Saglam S, Uludag V, Karaduman ZO, Arıcan M, Yücel MO, Dalaslan RE. Comparative evaluation of artificial intelligence models GPT-4 and GPT-3.5 in clinical decision-making in sports surgery and physiotherapy: a cross-sectional study. *BMC Med Inform Decis Mak* (2025) 25:163. doi: 10.1186/s12911-025-02996-8

10. Rosen J, Russell J, Kartik P, Vella‐Baldacchino M. Artificial intelligence algorithms in orthopaedics: A narrative review of methods and clinical applications. *J exp orthop* (2025) 12:e70549. doi: 10.1002/jeo2.70549

11. Xing Q, Xing X, Guo P, Tang Z, Shen Y. LLM-FMS: A fine-grained dataset for functional movement screen action quality assessment. *PLoS ONE* (2025) 20:e0313707. doi: 10.1371/journal.pone.0313707

12. Vivekanantha P, Cohen D, Slawaska-Eng D, Nagai K, Tarchala M, Matache B, Hiemstra L, Longstaffe R, Lesniak B, Meena A, et al. Evaluating the performance of five large language models in answering Delphi consensus questions relating to patellar instability and medial patellofemoral ligament reconstruction. *BMC Musculoskelet Disord* (2025) 26:1022. doi: 10.1186/s12891-025-09227-1

13. Liu Y, Li H, Ouyang J, Xue Z, Wang M, He H, Song B, Zheng X, Gan W. Evaluating Large Language Models for Preoperative Patient Education in Superior Capsular Reconstruction: Comparative Study of Claude, GPT, and Gemini. *JMIR Perioper Med* (2025) 8:e70047–e70047. doi: 10.2196/70047

14. Puce L, Bragazzi NL, Currà A, Trompetto C. Harnessing Generative Artificial Intelligence for Exercise and Training Prescription: Applications and Implications in Sports and Physical Activity—A Systematic Literature Review. *Applied Sciences* (2025) 15:3497. doi: 10.3390/app15073497

15. Diniz P, Grimm B, Mouton C, Ley C, Andersen TE, Seil R. High specificity of an AI-powered framework in cross-checking male professional football anterior cruciate ligament tear reports in public databases. *Knee Surg Sports Traumatol Arthrosc* (2025) 33:3478–3488. doi: 10.1002/ksa.12571

16. Safran E, Yildirim S. A cross-sectional study on ChatGPT’s alignment with clinical practice guidelines in musculoskeletal rehabilitation. *BMC Musculoskelet Disord* (2025) 26:411. doi: 10.1186/s12891-025-08650-8

17. Zhang L, Wang T, Zheng Y, Kong X, Hong G, Zang L. Assessment of ChatGPT’s adherence to evidence-based clinical practice guidelines for plantar fasciitis management. *J Orthop Surg Res* (2025) 20:434. doi: 10.1186/s13018-025-05831-y

18. Zhu X, Gao Z, Wang XA. Full-Parameter Fine-Tuning Method of LLMs for Sports Injury Prevention and Treatment. *International Journal of Mobile Computing and Multimedia Communications* (2025) 16: doi: 10.4018/IJMCMC.376486

19. Gültekin O, Inoue J, Yilmaz B, Cerci MH, Kilinc BE, Yilmaz H, Prill R, Kayaalp ME. Evaluating DeepResearch and DeepThink in anterior cruciate ligament surgery patient education: ChatGPT‐4o excels in comprehensiveness, DeepSeek R1 leads in clarity and readability of orthopaedic information. *Knee surg sports traumatol arthrosc* (2025) 33:3025–3031. doi: 10.1002/ksa.12711

20. Wang S, Bai Z, Gai Y. Toward intelligent clinical support for personalized sport training rehabilitation via large language models. *Health Inf Sci Syst* (2025) 14:17. doi: 10.1007/s13755-025-00416-9

21. Beda N, Mummareddy H, Qiu R, Porter E. Evaluation of artificial intelligence-generated responses to patient inquiries for orthopaedic sports procedures. *Journal of Orthopaedic Reports* (2026)100913. doi: 10.1016/j.jorep.2026.100913

22. King BW, Seilern Und Aspang J, Hammond K, Hill D, Jayaram P, Patel J, Danilkowicz RM. Evaluating Artificial Intelligence-Generated Responses to Patient Questions Regarding Orthobiologic Injections. *Orthopaedic Journal of Sports Medicine* (2026) 14:23259671251414852. doi: 10.1177/23259671251414852

23. Hsu W-K, Chuang H-C, Wang Y-Y, Hsu K-L, Kuan F-C, Su W-R, Chang C-H, Hong C-K. Concordance of ChatGPT, Gemini, Claude, and OpenEvidence with the 2024 AAOS guidelines on acute isolated meniscal pathology. *The Knee* (2026) 61:104427. doi: 10.1016/j.knee.2026.104427

24. Miller K, Sturm S, Dean K, Brochu B, Kassira W, Thaller S, Habal MB. Sport-Specific Craniofacial Injury Risk Stratification in Squash, Badminton, and Tennis Using NEISS and ChatGPT: A Structured Vignette Study. *J Craniofac Surg* (2025) 37:797–802. doi: 10.1097/SCS.0000000000012095

25. Ko S, Lee J, Ko K, Kim J. Benchmarking Open-Source Vision Language Models in Orthopedic In-Training Examination: A Comparison with Residents, Domain-Specific Evaluation, and Parameter Scaling. *Clin Orthop Surg* (2026) 18:159. doi: 10.4055/cios25183

26. Halvorson RT, Keeley T, Niknam K, Zack T, Majumdar S, Feeley BT, Zhang AL, Lansdown DA. Large Language Model Predicts Surgeon Recommendations for Imaging and Surgery for Patients Presenting for Knee and Shoulder Complaints With 70% and 81% Accuracy Using Previsit Questionnaire Responses. *Arthroscopy* (2026) 42:185–193. doi: 10.1002/arj.70016

27. Bandara E, Gore R, Shetty S, Mukkamala R, Rhea CK, Samulski BS, Hass A, Yarlagadda A, Kaushik S, De Silva M, et al. Standardization of Neuromuscular Reflex Analysis—Role of Fine-Tuned Vision-Language Model Consortium and OpenAI gpt-oss Reasoning LLM-Enabled Decision Support System. *Biomechanics* (2026) 6:23. doi: 10.3390/biomechanics6010023
