# Supplementary material for: Applications of natural language processing and large language models in sports injury assessment and rehabilitation decision-making: a scoping review
Source: Front Med (Lausanne). 2026 Jul 6;13:1866874. doi: 10.3389/fmed.2026.1866874 (PMC13382509; doi:10.3389/fmed.2026.1866874)
Supplement: Supplementary file 2 [file Table_2.docx]

**Appendix 2. Summary statistics of MINIMAR criteria-quality of reporting**

| **Key attributes** | **Value** |
| --- | --- |
| **Study population and setting** | **100%** |
| Population | 23(100.0%) |
| Study setting | 23(100.0%) |
| Data source | 23(100.0%) |
| Cohort selection | 23(100.0%) |
| **Patient demographic characteristics** | **4.35%** |
| Age | 3(13.0%) |
| Sex | 2(8.7%) |
| Race | 0(0.0%) |
| Ethnicity | 0(0.0%) |
| Socioeconomic status | 0(0.0%) |
| **Model architecture** | **78.26%** |
| Model output | 23(100.0%) |
| Target user | 23(100.0%) |
| Gold standard | 23(100.0%) |
| Model task | 23(100.0%) |
| Model architecture | 23(100.0%) |
| Features | 23(100.0%) |
| Data splitting | 4(17.4%) |
| Missingness | 2(8.7%) |
| **Model evaluation** | **61.96%** |
| External validation | 2(8.7%) |
| Transparency | 23(100.0%) |
| Optimization | 9(39.1%) |
| Internal validation | 23(100.0%) |
| the overall adherence rate | 61.70% |
